# Reference-based RADseq resolves robust relationships among closely related species of lichen-forming fungi using metagenomic DNA

**DOI:** 10.1038/s41598-017-09906-7

**Published:** 2017-08-29

**Authors:** Felix Grewe, Jen-Pen Huang, Steven D. Leavitt, H. Thorsten Lumbsch

**Affiliations:** 1 Integrative Research Center, Science and Education, Field Museum of Natural History, 1400S Lake Shore Drive, Chicago, IL 60605 USA; 2 Department of Biology & M. L. Bean Life Science Museum, Brigham Young University, Provo, UT 84602 USA

## Abstract

Despite increasing availability of phylogenomic datasets, strategies to generate genome-scale data from organisms involved in symbiotic relationships remains challenging. Restriction site-associated DNA sequencing (RADseq) can effectively generated reduced representation genomic loci. However, when using metagenomic DNA from inseparable symbiotic organisms, RADseq loci may belong to any number of the organisms involved in these intimate associations. In this study, we explored the potential for a reference-based RADseq approach to generate data for lichen-forming fungi from metagenomic DNA extracted from intact lichens. We simulated RAD data from draft genomes of closely related lichenized fungi to test if RADseq can reconstruct robust evolutionary relationships. Subsequently, we generated empirical RADseq data from metagenomic lichen DNA, with RADseq loci mapped back to a reference genome to exclude loci from other lichen symbionts that are represented in metagenomic libraries. In all cases, phylogenetic reconstructions using RADseq loci recovered diversification histories consistent with a previous study based on more comprehensive genome sampling. Furthermore, RADseq loci were found to resolve relationships among closely related species, which were otherwise indistinguishable using a phylogenetic species recognition criterion. Our studies revealed that a modified, reference-based RADseq approach can successfully be implemented to generate symbiont-specific phylogenomic data from metagenomic reads.

## Introduction

The continued development of high-throughput sequencing approaches enables time and cost efficient generation of massive amounts of genomic data and has facilitated the expansion of genomic datasets for phylogenetic- and population-level studies from single- and multi-locus matrices to genetic data sampled across entire genomes^1^. A wide range of methods are commonly used to generate genome-wide datasets, but rather than focusing on sequencing complete genomes, most methods are designed to sample subsets of organisms’ genomes. Direct sequencing approaches, e.g. RNAseq^2, 3^ or genome skimming of highly abundant genomic regions or organelle genomes^4, 5^, and targeted approaches using baits to either pull genes of interest^6, 7^ or target ultra-conserved elements^8^, can effectively generate reduced representation genome-scale datasets.

Among the most efficient reduced representation sequencing methods is restriction associated DNA sequencing (RADseq). In the original RADseq protocol, restriction enzymes were used to digest genomic DNA, and the resulting fragments are then sheared to sizes appropriate for a high-throughput sequencing^9, 10^. Today, a plethora of RADseq techniques exist that vary from this original protocol, all of which share the common feature of sequencing loci flanking conserved restriction enzyme recognition motifs^11^. This flexibility allows RADseq protocols to be adjusted to best fit different genome sizes by the choice and number of restriction enzymes, thereby fragmenting genomic DNA to various degrees^12^. In addition, most protocols add a size-selection step to reduce the number of fragments that are sequenced so that a sufficient depth of coverage is obtained. While some initial knowledge about the targeted genome can be helpful (e.g. size and G/C content to determine the most appropriate restriction enzyme), RADseq can be applied without knowledge of a reference genome, hence is an ideal method for non-model organisms.

Based on its relatively uncomplicated setup and low costs, RADseq has been applied to the study of a wide range of organisms. RADseq has been used for both model and non-model organisms, and has answered a wide variety of biogeographical, ecological, evolutionary, and conservation-related questions^13–20^. RADseq has also been shown to successfully delimit species and reconstruct phylogenies of both recently diverged groups^21–24^ and clades with diversifications histories spanning tens of millions of years^25–27^.

Although RADseq is increasingly popular since its development, with over 600 publications mentioning any of the RADseq variations in the year 2016^11^, this approach has rarely been used to study organisms involved in intimate, largely inseparable symbiotic relationships. Implementing RADseq protocols for these symbiotic organisms is challenging since the sequencing pool harbors metagenomic DNA from individuals from evolutionarily distinct lineages. A limited number of studies of symbiotic organisms using RADseq have circumvented this problem by either growing the targeted organism in axenic cultures or physically separating symbiotic partners by hand^28, 29^. However, for many organisms growing in intimate symbiotic associations, such as lichen-forming fungi, it is difficult to physically separate the symbiotic partners and growth of axenic cultures may be prohibitively slow^30, 31^. In these cases, a reference-guided approach is crucial to separate the metagenomic pool of sequences that was derived from all symbiotic partners of the holobiont.

Lichens provide interesting systems for studying diversification in obligate symbiotic systems^32–34^. Developing time and cost-effective approaches for generating genome-scale datasets is crucial to infer robust diversification histories and gain novel insight into evolutionary processes in these symbiotic fungi. However, to-date only a limited number of studies generated genome-scale datasets for inferring phylogenetic relationships^35, 36^. A reference-guided whole-genome skimming approach was used to generate various phylogenomic datasets for a clade of closely-related lichen-forming fungal species by mapping raw metagenomic sequence reads to a reference genome sequenced from an axenic fungal culture^36^. Although this study was able to infer robust phylogenetic relationships, the genome skimming approach was relatively costly and limited to few dozen individuals. It remains unclear how other reduced representation approaches for generating phylogenomic datasets can effectively be applied to metagenomic DNA pools isolated from lichen holobionts.

The *Rhizoplaca melanophthalma* species complex provides a useful study system to investigate if a reference-guided RADseq approach can successfully be implemented to generate meaningful phylogenomic data for the fungal partner from metagenomic lichen DNA. This species complex is comprised of nine species – *R*. *haydenii*, *R*. *idahoensis*, *R*. *melanophthalma*, *R*. *novomexicana*, *R*. *occulta*, *R*. *parilis*, *R*. *polymorpha*, *R*. *porteri*, and *R*. *shushanii* ^37^, which diversified largely during the Pliocene and into the Pleistocene^38^. This clade of closely related lichen-forming fungi is relatively well-studied^39^, and crucial genomic and phylogenomic resources are available for comparisons, including a draft genome assembly based on an axenic fungal culture and other genome-scale datasets^36^. A number of species in this complex have broad, intercontinental distributions, and genome-scale data will be crucial to better understand population structure and dispersal capacity of these cosmopolitan species. In contrast, other species, including western North American endemics *R*. *haydenii* and *R*. *idahoensis*, have geographically restricted distributions and populations are threatened by habitat alteration due to agriculture, grazing, and invasive species^40^. Finally, relationships and boundaries among a number of closely related species, *R*. *occulta*, *R*. *polymorpha*, and *R*. *porteri* – the ‘porteri group’, remain unresolved^36^.

In this study, we investigated the utility of RADseq for generating symbiont-specific phylogenomic datasets from metagenomic samples, such as symbiotic lichens. RADseq loci from lichen metagenomes were sorted by a mapping approach against a reference lichen-fungus genome sequence. We first tested if RADseq data can reconstruct evolutionary relationships within the *Rhizoplaca melanophthalma* multi-species complex consistent with those inferred from other, more comprehensive genome-wide datasets. For this study, we simulated RAD data from draft genome sequences from an earlier study. After successfully reconstructing a *Rhizoplaca* phylogeny from the simulated data and consistent with previous reconstructions, we generated empirical RADseq data from metagenomic lichen DNA and added the empirical RADseq data to the simulated data for a hybrid analysis. Finally, we investigated if the RADseq data can also be utilized for population genomics analysis to resolve shallow relationships in the *Rhizoplaca* phylogeny, investigating relationships in the ‘porteri group’. Our studies revealed that a modified RADseq approach can successfully be implemented to generate symbiont-specific phylogenomic data from metagenomic reads in a cost- and time-efficient method.

## Material and Methods

### Genomic data used in RADseq simulation

For the RADseq simulation approach, we utilized draft genome sequences from 30 specimens of the *R*. *melanophthalma* species complex from a previously published study and a reference genome generated from an axenic mycobiont culture of the lichenized fungi *Rhizoplaca melanophthalma sensu stricto* ^36^. The taxon sampling for the RADseq simulation included: *R*. *haydenii* (n = 2), *R*. *melanophthalma* (6), *R*. *novomexicana* (1), *R*. *occulta* (2), *R*. *parilis* (4), *R*. *polymorpha* (6), *R*. *porteri* (5), and *R*. *shushanii* (5).

### RADseq simulation

Since lichens are symbiotic associations consisting of evolutionarily independent lineages and DNA extracted from a lichen thallus is comprised of genomes from all associated symbionts, we separated the metagenomic data of each sample with a mapping approach to a reference fungal genome from a *R*. *melanophthalma* culture. In Leavitt *et al*.^36^, the reference genome sequence of this lichen fungus was *de novo* assembled by using the RAY v2.3.1 assembler^41, 42^ with a kmer value of 41, and included all scaffolds with sizes larger than 5 kb for subsequent analyses. The total DNA of the symbiont genomes of the remaining taxa were sequenced with a low coverage, which allowed sorting of the reads by mapping them onto the reference sequence. Within the RealPhy v1.10 pipeline^43^, raw reads of the low-covered 30 taxa were mapped to the reference genome with Bowtie2 v2.1.0^44^. RealPhy was used with the following arguments: -readLength 75 -perBaseCov 5 -gapThreshold 0.2. An average of 70.8% of the reference genome was covered by the mapping the metagenomes. A consensus sequence of the successfully mapped reads was extracted for each specimen and used for the RADseq dataset simulation.

RADseq datasets of the *de novo* reference genome of *R*. *melanophthalma sensu stricto* and the 30 consensus sequences of the *R*. *melanophthalma* species complex were simulated by using the R package SimRAD^45^. After an initial test using different cut sites on the reference sequence to select for the best restriction enzyme, we *in silico* digested all 30 genome sequences with ApeKI (5-cutter with one ambiguous position: G|CWGC). The digested genome was subsequently selected for fragments with sizes between 200 and 500 bp. From these size-selected fragments, the first and last 143 bp sequences were extracted simulating a 150 bp Illumina sequencing after adapter and barcode sequences were removed. Since loci that are overlapping by more than 30% are merged in the pyRAD pipeline, we extracted the full sequence of the size-selected fragments instead of two separate sequences when the fragments were shorter than 243 bp.

**Figure 1:**
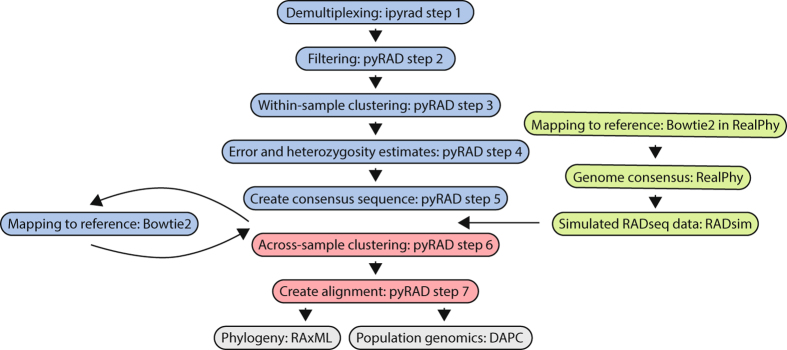
Computational workflow of analysis. Green boxes in the flow chart show the treatment of the simulated data before adding it to pyRAD (steps 6 and 7, in red) followed by a phylogenetic analysis (grey). Blue boxes in the flow chart represent the initial processing of the empirical data in ipyrad and pyRAD with an additional step of lichen fungus identification by mapping the metagenomic loci to the reference sequence. The mapped loci were then added to the simulated data (green) for a combined processing in pyRAD (steps 6 and 7, in red) prior to phylogenetic and population genomics analyses (grey).

The output was formatted to fit into the pyRAD v3.0.64^46^ pipeline after step 5 when raw reads have been demultiplexed (step 1), quality filtered (step 2), within-sample clustered (step3), error and heterozygosity estimated (step 4), and have had consensus sequences (loci) created (step 5) (Fig. 1). The simulated data – representing the consensus sequences of each cluster (loci) – was across sample clustered in the pyRAD pipeline with vsearch^47^ set to a 90% clustering threshold (Wclust = .90) (step 6). Finally, all clusters were filtered for a final alignment (step 7) that required a minimum of four samples for each final locus (MinCov = 4). The data type was set to ‘gbs’ since all fragments have an ApeKI cut site on both ends, which would qualify for sequencing from either direction. The resulting alignments of unlinked loci (.unlinked_snps) were used for phylogenetic analysis.

### Genomic data used for empirical RADseq in the laboratory

For an empirical comparison using genomic data generated by RADseq lab procedures, we selected 57 additional specimens of the *Rhizoplaca melanophthalma* species complex. All specimens were collected from sites throughout western North America (Supplementary table 1). Total metagenomic DNA was extracted either by following a modified CTAB protocol^48^ or by using the Prepease DNA Isolation Kit (USB – product discontinued). Prior to the preparation of the RADseq libraries, we verified the identity of each specimen by sequencing their nuclear ITS rDNA region with a combination of primers ITS1f^49^ and ITS4^50^ and a comparison to a previously published worldwide sampling^37^.

### RADseq library production and sequencing in the laboratory

RADseq libraries were prepared as described earlier^51^ with an additional size selection step. In short, for the RADseq library production 100 ng of each DNA isolation was dried overnight together with approximately 0.06 pmol of adapters (designed as outlined elsewhere^52^), then resuspended for a digestion with the restriction enzyme ApeKI (New England Biolabs). This resuspension was followed by a ligation using a T4 ligase (New England Biolabs) as described^51^. Up to 48 samples with compatible barcodes were pooled and selected for fragments of sizes between 300 and 500 bp using the BluePippin (Sage Science). The pooled libraries were amplified using the REDTaq ReadyMix (Sigma-Aldrich) with primer pairs that were binding the ligated adapters (see ref. 52 for sequence details). The completed libraries were directly sequenced on an Illumina MiSeq using the MiSeq Reagent Kit v3 for 150 cycles (Illumina) to produce single-end sequences with a length of 150 bp.

### Combined analysis of simulated and empirical RADseq datasets

The raw reads from two MiSeq runs were individually demultiplexed in step one of the ipyrad workflow (https://github.com/dereneaton/ipyrad/blob/master/docs/index.rst) (Fig. 1). Subsequently, steps two to five were processed with pyRAD v3.0.64^46^ for genotype-by-sequencing data (Datatype = gbs) of haploid genomes (ploidy = 1). Within-sample clustering was done by vsearch^47^ set to a 90% clustering threshold (Wclust = 0.90). The consensus sequence was generated from all clusters with a minimum coverage of four reads (Mindepth = 4). These consensus sequences – representing all the recovered metagenomic loci from each sample – were selected for sequences of lichen fungal origin by mapping them to the reference sequence with Bowtie2 with adjusted parameters to one permitted mismatch (−N 1), a seed length of 20 (−L 20), up to 20 seed extension attempts (−D 20), and a maximum “re-seeding” of 3 (−R 3). The mapped loci were then combined with the simulated data for an across sample clustering in pyRAD (step 6 and step 7) with the same parameter setting as used for only simulated data (see above; Wclust = 0.90; MinCov = 4). Samples for which the final statistics output indicated less than 5000 loci were removed for a repeated run of step 6 and step 7. In an additional analysis, we lowered the missing data in the final alignment by increasing the minimum number of samples per final loci (MinCov = 30). Phylogenetic analyses were done with the resulting alignments that only included the unlinked loci (.unlinked_snps) of the combined data from empirical and simulated samples.

Correlation of data from samples of simulated and empirical origin was tested by a linear regression model in R^53^. The proportion of shared loci of the combined simulated and empirical data were generated in a pairwise similarity matrix and displayed by using the R package RADami^25^.

### Phylogenetic Analysis of RADseq datasets

Phylogenetic trees were estimated using maximum likelihood interference with RAxML v7.2.8^54^ using the GTR + G + I model. For each analysis, 100 bootstrap replicates were calculated using the fast bootstrapping option implemented in RAxML^55^. Trees were drawn with the TreeExplorer implemented in MEGA 7.0.20^56^. Based on previous studies, all resulting trees were rooted with *R*. *novomexicana*.

**Figure 2:**
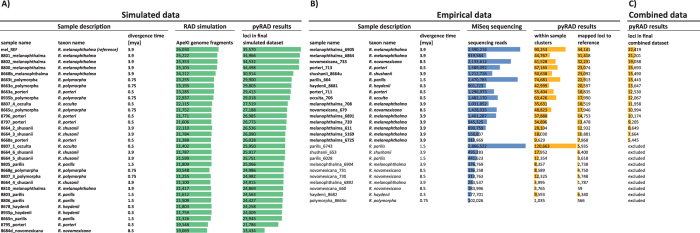
Overview of the RADseq results after individual steps of analyses. (**A**) Number of fragments and loci in the final dataset of the simulation. (**B**) Number of raw sequences, within sample clusters (after pyRAD step 5), and mapped loci to the reference sequence of the empirical dataset. (**C**) Number of loci in the final combined dataset (after final pyRAD step 7). Bars represent the respective values in relation to their colored group. Divergence times were used according to Leavitt *et al*.^38^. Additional 31 samples with less than 20,000 sequences were excluded (not shown in table). Samples in bold had a total number of more than 5,000 loci in the final dataset and were included for further analyses.

The genetic structure of the members of the ‘porteri group’ was evaluated with the Discriminant Analysis of Principal Components (DAPC) implemented in the R package adegenet v2.0.1^57, 58^. This method relies on a data transformation by a Principal Component Analysis (PCA) prior to the separation of individuals of a population by their genetic distance using a Discriminant Analysis (DA). To minimize the missing data for the PCA, we treated the dataset by using the “mean” option for genotypic data type as recommended^57^ and excluded loci that had more than 20% missing data. For the DAPC, the data was loaded into an R data frame with settings for a haploid genome (ploidy = 1). The DAPC was then conducted by using the first five principal components and all (two) DA-eigenvalues. In addition to the display of the genetic variation in the genomic space, the DAPC allows a prediction of the group membership probability for each sample. Group memberships for samples were predefined according to the corresponding ‘taxon name’ in Fig. 2.

### Reproducibility

All scripts that were used in this study are available (https://github.com/felixgrewe/lichen_RADseq). All RAD sequences were deposited in the NCBI Sequence Read Archive (SRA) with accession numbers SRR5807616 - SRR5807612.

## Results and Discussion

### RADseq simulation

For the RADseq simulation, the reference genome sequence of *R*. *melanophthalma* was *in silico* digested by six different restriction enzymes (SbfI, PstI, NsiL, BclI, BstYI, and ApeKI). The resulting fragments of sizes between 200 and 500 bp were counted to identify the best restriction enzyme for this study (Supplementary Fig. 1). The lichen-fungal genome of *R*. *melanophthalma* has a small size of 38.7 Mb^36^, which demands the use of a common-cutting restriction enzyme that produces a sufficient number of fragments for the RADseq analysis. The simulated use of 6- and 7-cutter restriction enzymes produced an insufficient number of fragments for RADseq (0–1,173 fragments, 3,463 fragments when the recognition motif of the 6-cutter had two ambiguous sites). However, we highly increased the fragment number to 26,030 by cutting the genome with ApeKI – a 5-cutter with one ambiguous site in its recognition motif. We then selected ApeKI for all further analyses, since it produced enough fragments to represent sufficient genomic variation of the relatively small lichen-forming fungal genomes – ca. 40 Mb – and compensate for a potential loss of homologous loci due to allele drop-out (the loss of loci due to mutations in the enzyme recognition motif.)

Between 19,069 and 25,222 fragments were produced using the restriction enzyme ApeKI on each of the *Rhizoplaca* genomes in the simulation (Fig. 2A). These fragments were integrated into the pyRAD pipeline (after step 5, Fig. 1) and across sample clustered into 13,434 to 35,570 loci per sample. The final alignment contained 53,659 positions and 56.77% gaps.

**Figure 3:**
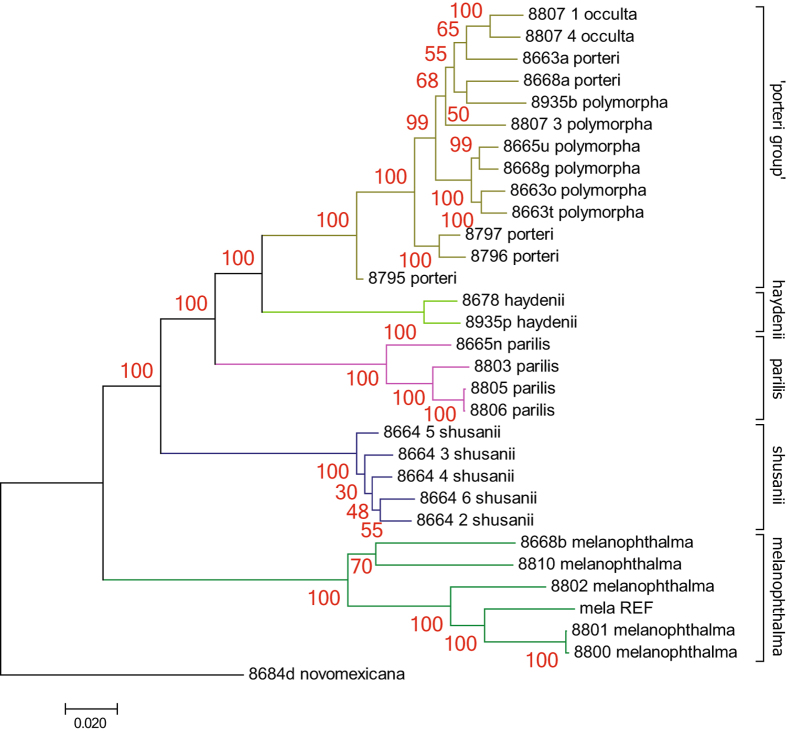
Phylogenetic tree inferred from the simulated RAD data. Samples representing each species are highlighted with individual branch colors. Bootstrap values are indicated near nodes in red. The unit of the branch lengths is substitutions per site.

The phylogeny inferred from the simulated RAD loci (Fig. 3), representing a sub-sampling of the genomic data to a reduced genome representation, recovered identical phylogenetic relationships among species compared to the most comprehensive phylogenomic dataset – ‘RealPhy’ – in Leavitt *et al*.^36^. All species of the *Rhizoplaca melanophthalma* species complex were recovered as individual monophyletic groups supported by strong bootstrap support except for the ‘porteri group’, which included *R*. *polymorpha*, *R*. *porteri*, and *R*. *occulta*. To extend the current phylogeny, we generated further RAD loci from more individuals in the laboratory and incorporated them into the simulated dataset for a combined processing of simulated and empirical RAD loci using pyRAD.

### RAD sequencing

For the RAD library production in the laboratory, we built two libraries of total lichen metagenomic DNA isolations. These libraries were sequenced by a total of 17,7 × 10^6^ and 19,8 × 10^6^ reads, respectively. The number of reads of each successfully sequenced sample (>20,000 reads) varied widely, from 102,026 to 2,8 × 10^6^ reads with an average of 1,03 × 10^6^ (sd = 794,051) reads per sample (Fig. 2B). This variation is most likely caused by an uneven normalization of the libraries prior to sequencing. A proper normalization of the libraries is an important step which avoids overrepresentation of a few samples and warrants maximum efficiency from the sequencing. The number of within sample clusters that pyRAD generated from these sequences directly correlated with the initial number of sequences (R^2^ = 0.9295, not shown) indicating that the higher the initial sequencing, the more clusters were generated.

A crucial step in this pipeline was the identification of the fungal clusters from the pool of metagenomic within-sample clusters by mapping them to the lichen-fungus genome of *R*. *melanophthalma*. The mapping detected that an average of 43% (sd = 18%) of the clusters originated from the fungal genome leaving between 59 to 34,141 loci for each sample in the across-sample clustering step (Fig. 2B). Therefore, in most metagenomic pools of samples the number of lichen-fungal loci were smaller than the number of other loci from an unidentified origin. Multiple studies reported that lichens consist of more than just mycobiont and photobiont genomes, but can also harbor multiple algae species^59–61^, high content of bacteria^62–65^, and/or even additional, distantly related, fungi^32^. Any of these organisms could have contributed to the metagenomic pool and increased the amount of non-lichen fungal sequences. With an increasing number of potential reference genomes for these organisms available in Genbank, the identification of these loci of unknown origin could be integrated into interesting future studies.

Two samples differed from the general observation that around half of the metagenomic pool has fungal origin. The *R*. *parilis* sample ‘parilis_6743’ had most raw reads (2,9 × 10^6^) and most metagenomic clusters (120,663) of all samples, but only 5,935 of these clusters (5%) consisted of fungal DNA. Since this pattern was not consistent throughout all the *R*. *parilis* samples, these numbers may reflect contamination or misidentification of the sample. In contrast, the *R*. *melanophthalma* sample ‘melanophthalma_6725’ contained over 82% fungal clusters. Hence, it could be included in the final dataset although the sample only had a relatively small number of initial raw sequences.

On average, 67% (sd = 2.9%) of the fungal loci successfully across-sample clustered with loci from at least three other samples (Fig. 2C). After this clustering, a total of 47 samples remained with more than 5,000 loci. These samples were included in the final dataset of 76,371 loci and a final alignment of 67,775 positions with 70.46% gaps.

### Correlation of simulated and empirical RAD data

**Figure 4:**
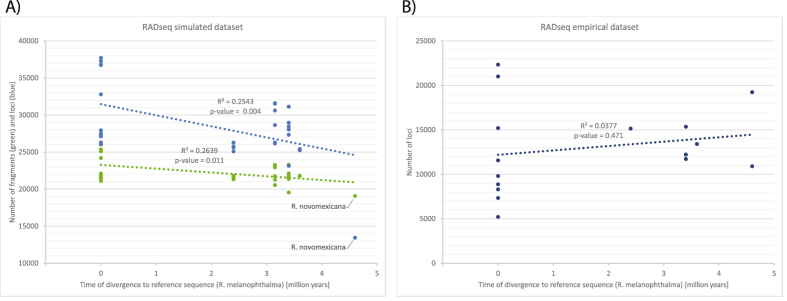
Correlation of RADseq results to the phylogenetic distance of the samples to the reference sequence (*R*. *melanophthalma*). (**A**) The number of fragments and loci of the final simulated dataset are shown in green and blue, respectively. (**B**) The number of loci of the empirical RADseq samples are indicated in orange. All numbers are plotted against the time of divergence of samples to the reference, *R*. *melanophthalma*. Trendlines are based on the linear regression model and are drawn in the respective colors of the different datasets.

For the simulated dataset, reads were initially mapped to the reference to create a consensus sequence of the lichen-fungal genome for each sample. The number of RAD fragments and loci that were recovered from lichen metagenomes was dependent on the distance of the relationship between samples and the reference genome that was targeted (Fig. 4A). The relationship of the species had less effect on the mapping, represented here by the number of fragments after a simulated digest (R^2^ = 0.2639, p = 0.011), rather than on the number of final loci produced by the pyRAD pipeline (R^2^ = 0.2543, p = 0.004). This correlation also remained significant after excluding the outgroup *R*. *novomexicana* for the fragments (R^2^ = 0.2093, p = 0.011) and loci (R^2^ = 0.1805, p = 0.019). For *R*. *novomexicana*, which is most distantly related to the reference, the number of loci dropped much lower than projected by the regression model. The drop of loci cannot be caused by allele dropout since the simulated digest produced fragment numbers close to the prediction. However, it demonstrates that the mapping of the raw reads is less sensitive (allows more mapping) than the cluster generation in pyRAD under the current parameter settings. The processing of more distantly related taxa would therefore require an adjustment of the clustering threshold, which also increases the risk of clustering paralogous and non-homologous loci. The mapping approach implemented in ‘RealPhy’ may be less effected by genomic variation and therefore suitable to recover more loci from distantly related taxa. However, more distantly related individuals would be needed in this study to further support this observation.

**Figure 5:**
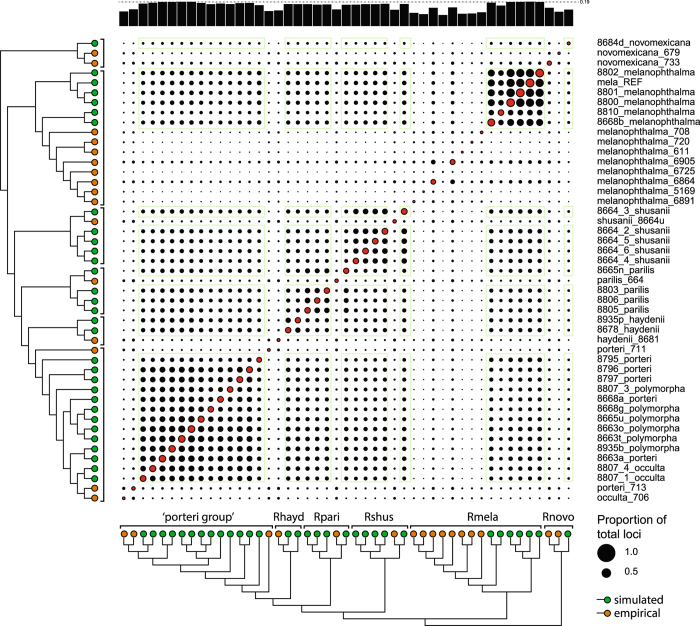
Proportion of shared loci among individuals of simulated and empirical origin. Correlation plot shows percentage of shared loci and successfully amplified loci for each sample in black and red circles, respectively. Green boxes comprise correlations of samples derived from simulated data. Black bars on top of the plot indicate the average percentage of shared loci per sample. The phylogenetic trees were calculated from the combined dataset as in Fig. 5 with green and orange tips indicating samples derived from simulated and empirical data, respectively.

For the empirical dataset, reads were first within sample clustered in pyRAD before they were sorted by mapping to the fungal reference sequence. The number of final loci of the empirical dataset is not correlated with an increasing distance of the species relationship (Fig. 4B) and differed from the projection based on the simulated dataset (Fig. 4A). This difference indicates that the variation in loci number results from biases during the RADseq laboratory procedures rather than from errors in the subsequent computational sequence analysis. A lower number of loci also leads to fewer shared loci across the empirical samples compared to simulated samples as indicated in a pairwise comparison of the data (Fig. 5).

### Phylogenetic analysis of combined data

**Figure 6:**
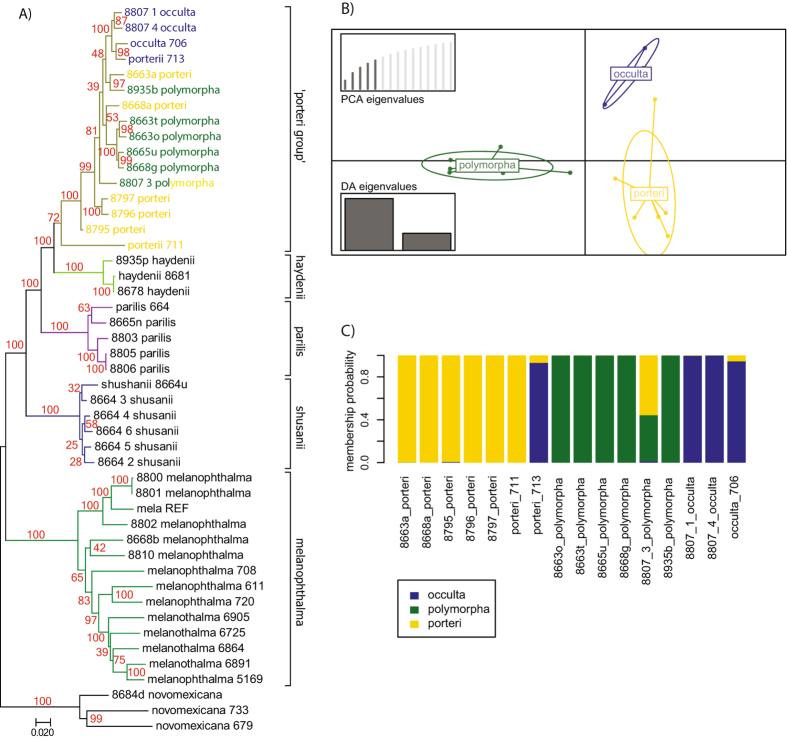
Relationship of the *R*. *melanophthalma* species complex estimated from the combined dataset. (**A**) Phylogenetic tree inferred from the combined dataset. Samples representing each species are highlighted with branch colors as in Fig. 2. Bootstrap values are represented by red numbers near nodes. The unit of the branch lengths is substitutions per site. The color of the label of the samples of the ‘porteri group’ show the group membership probability estimated with the DAPC. (**B**) DAPC scatterplot of samples of the ‘porteri group’. Individuals and groups are represented by dots and inertia ellipses, respectively. (**C**) STRUCTURE-like plot of group membership probabilities of samples of the ‘porteri group’.

Phylogenetic analyses of the combined dataset recovered all species of the *Rhizoplaca* species complex as monophyletic groups, except for those within the ‘porteri group’, in which the species *R*. *occulta*, *R*. *polymorpha*, and *R*. *porteri* were recovered intermixed in a single, well-supported clade (Fig. 6A). The tree topology is therefore similar to inferences from the simulated RAD data (Fig. 3) and the whole-genome dataset used in Leavitt *et al*.^36^. In this topology, all newly included 16 samples clustered within their respective species groups, and the monophyly of each group remained strongly supported, with the exception of those in the ‘porteri group’. This group remained unresolved even after the addition of new samples. Moreover, the support for the monophyly of the ‘porteri group’ decreased (bootstrap = 72) largely due to the addition of specimen ‘porteri_711’, which was resolved as sister to all remaining group taxa. Using a smaller alignment (at least 30 samples/loci, 7770 positions, 27.1% gaps), specimen ‘porteri_711’ was recovered as sister to the ‘porteri group’ and *R*. *haydenii*, which explains that the weaker bootstrap support in both trees is due to its unstable position (Supplementary Fig. 2). After including new samples, another difference occurred within the ‘porteri group’ where the added specimen ‘porteri_713’ clustered together with the remaining *R*. *occulta* individuals and disrupted the former monophyly of the group.

Since the ‘porteri group’ remained unresolved by the maximum likelihood tree reconstruction, we tested population genetic approaches using the RAD data generated here to differentiate the genomes by their variation. In preparation for the DAPC, we filtered the dataset on missing data which resulted in a total of 4997 qualified loci. The DAPC combines a PCA with a DA that supports a separation of genomes based on their variance between groups rather than their variance within groups. All three species of the ‘porteri group’ built clearly separated clusters supporting their genomic divergence from each other (Fig. 6B). In addition, the DAPC method exploits group memberships of individuals, which are represented by a STRUCTURE-like plot (Fig. 6C). In this analysis, all individuals of the ‘porteri group’ were assigned high probabilities to distinct groups corresponded to their nominal species, with the exception of specimen ‘8807_3_polymorpha’, which the membership probabilities were divided between *R*. *polymorpha* and *R*. *porteri*. All other *R*. *polymorpha* and *R*. *occulta* specimens belonged to their respective membership groups. Of the samples representing *R*. *porteri*, however, one specimen, ‘porteri_773’, was assigned membership to the *R*. *occulta* group with high probability. The same sample also clustered with high support within the *R*. *occulta* clade in the phylogenetic tree (Fig. 6A). Hence, the DAPC method may have identified a misidentified sample, under which condition the *R*. *occulta* would remain monophyletic as earlier observed (Fig. 3, ref. ^36^).

The combined RAD dataset was sufficient to infer well-resolved phylogenies. Even though some of the samples lacked variation and were not well separated in the phylogenetic trees, these samples could be distinctly separated by population genetic methods such as DAPC. Compared to other reduced genome representation methods such as sequence capturing, RADseq produces a higher number of loci with relatively little effort in the laboratory and much lower costs^66^. Therefore, future phylogenetic and population genetic analyses of lichen-fungal genomes may solely rely on reduced genome representation data, such as RADseq, which will dramatically reduce sequencing costs and allow a deeper (taxon-) sampling for each study.

## Conclusion

We successfully implemented a strategy for generating RADseq loci for targeted lineages involved in intimate symbiotic associations, lichen-forming fungi. Whole lichens were sequenced and reduced representation RADseq metagenomic libraries were filtered for loci derived from the lichen-fungal genome using a reference-guided mapping approach. The resulting reduced genome representation dataset had sufficient phylogenetic signal to reconstruct phylogenetic trees consistent with those using more comprehensive genome-scale datasets. A limiting factor of this reference-guided RADseq method is the requirement of a closely related reference genome. This requirement, however, will be less of a restraining factor in the future with an increasing number of complete lichenized fungal genomes available in Genbank. In addition, applications for metagenomic high-throughput sequencing are increasingly sophisticated for separating *de novo* in-depth sequenced metagenomes. These advances will allow for future studies to create reliable lichen-fungus reference sequences to sort metagenomic RAD loci. Hence, the low-cost RADseq method presented here can be applied for reduced genome representation of other lichen species. Thereby the opportunity to sample thousands of individuals in just one high-throughput sequencing run will open new avenues for lichen phylogenomic and population genomics analyses.

## Electronic supplementary material

Supplementary Material Online
