# Supplementary material for: Reference-based RADseq resolves robust relationships among closely related species of lichen-forming fungi using metagenomic DNA
Source: Sci Rep. 2017 Aug 29;7:9884. doi: 10.1038/s41598-017-09906-7 (PMC5575168; doi:10.1038/s41598-017-09906-7)

SUPPLEMENTARY MATERIAL

Reference-based RADseq resolves robust relationships among closely related species of lichen-forming fungi using metagenomic DNA

Felix Grewe[1], Jen-Pen Huang[1], Steven D. Leavitt[1,2], and H. Thorsten Lumbsch[1]

[1] Integrative Research Center, Science and Education, Field Museum of Natural History, 1400 S Lake Shore Drive, Chicago, IL 60605, USA.

[2] Department of Biology & M. L. Bean Life Science Museum, Brigham Young University, Provo, UT, 84602 USA.

SUPPLEMENTARY MATERIAL ONLINE

Supplementary Table 1 – Origin of samples used in for this study.

Supplementary Figure 1 – Simulation of genome fragmentation using different Restriction Enzymes. Distribution of fragment sizes based on the digestion by restriction enzymes SbfI, PstI, NsiL, BclI, BstYI, and ApeKI is shown by grey bars. The proportion of fragments of sizes between 200 and 500 bp is highlighted in red.

Supplementary Figure 2 – Phylogenetic tree inferred from the combined dataset that required at least 30 samples/loci for the final alignment. Bootstrap values are represented by red numbers near nodes. The unit of the branch lengths is substitutions per site.

Supplementary Table 1

DNA #	Clade (Leavitt *et al.* , 2011)	Voucher	Location	ITS acc. nos.	Latitude	Longitude	Elevation (m)	Collector(s)
shushanii_544	*R. melanophthalma* C3	BRY C55062	USA, Utah, Wayne Co.: Thousand Lake Mountain (9)	HM577283	38.4366	-111.4677	3270	LDP
shushanii_543	*R. melanophthalma* C4d	BRY C55103	USA, Utah, Wayne Co.: Thousand Lake Mountain (4)	HM577334	38.5079	-111.5161	2550	LDP
**melanophthalma_611**	*R. melanophthalma* C2	BRY C55044	USA, Utah, Wayne Co.: northwest of Boulder Mountain (BM-1)	HM577265	38.27364	-111.6106	2344	SDL, HCL, JHL, PAR
melanophthalma_612	*R. melanophthalma* C2	BRY C55045	USA, Utah, Wayne Co.: Thousand Lake Mountain (1)	HM577266	38.4243	-111.6446	2220	LDP
parilis_626	*R. melanophthalma* C4b	BRY C55080	USA, Utah, Wayne Co.: Thousand Lake Mountain (3)	HM577311	38.5079	-111.5505	2400	LDP
parilis_632	*R. melanophthalma* C4b	BRY C55081	USA, Utah, Wayne Co.: Thousand Lake Mountain (5)	HM577312	38.5076	-111.4904	2725	LDP
shushanii_653	*R. melanophthalma* C3	BRY C55070	USA, Utah, Wayne Co.: Thousand Lake Mountain (9)	HM577291	38.4366	-111.4677	3270	LDP
porteri_658	*R. melanophthalma* C4d	BRY C55570	USA, Utah, Wayne Co.: Thousand Lake Mountain (10)	HM577376	38.4432	-111.4703	3400	LDP
novomexicana_660	*R. melanophthalma* C2	BRY C55048	USA, Utah, Wayne Co.: Thousand Lake Mountain (10)	HM577269	38.44317	-111.4703	3400	LDP
**parilis_664**	*R. melanophthalma* C4b	BRY C55088	USA, New Mexico, San Juan Co.: vicinity of Aztec Ruins National M	HM577319	36.8348	-108.0002	1721	SDL, HCL
polymorpha_669	*R. melanophthalma* C4c	BRY C55094	USA, Idaho, Owynee Co.: McBride Creeks Badlands	HM577325	43.3202	-116.9795	1291	SDL, HCL, JHL
**novomexicana_679**	*R. melanophthalma* C2	BRY C55050	USA, Utah, Emery Co.: San Rafael Swell	HM577271	38.70424	-110.7964	1967	SDL
parilis_692	parilis	EA 15-123A (BRY-C)	USA, NV, Elko Co., Humboldt National Forest, Jarbridge WA, switc	KU934750	-	-	-	-
occulta_695	*R. melanophthalma* C4a	BRY C55074	USA, Utah, Juab Co.: West of Goshen	HM577305	39.9697	-112.0601	1840	LLS (EA 18-140)
parilis_698	*R. melanophthalma* C4b	BRY C55089	USA, Montana, Deer Lodge Co.: southwest of Anaconda Copper S	HM577320	46.0565	-112.982	1890	LLS (EA 21-166
**occulta_706**	*R. melanophthalma* C4a	BRY C55075	USA, Idaho, Butte Co.: Salmon Challis National Forest	HM577306	43.7197	-113.0891	2432	LLS (EA 37-356)
**melanophthalma_708**	*R. melanophthalma* C2	BRY C55055	USA, Idaho, Lemhi Co.: Salmon Challis National Forest	HM577276	44.56022	-113.3507	1194	LLS (EA 41-403)
**porteri_711**	porteri (aff.)	Leavitt 711 (BRY-C)	USA, NV, White Pine Co.: Humboldt-Toiyabe N.F. Kalamazoo Mtn	NA	38.54642	-114.6385	2744	LLS, SDL, JS
porteri_712	porteri (aff.)	Leavitt 712 (BRY-C)	USA, NV, White Pine Co.: Humboldt-Toiyabe N.F. Kalamazoo Mtn	KU934836	38.54642	-114.6385	2744	LLS, SDL, JS
**porteri_713**	*R. melanophthalma* C4d	BRY C55149	USA, Nevada, White Pine Co.: Humboldt-Toiyabe National Forest	HM577380	38.5464	-114.6385	2744	SLD, LLS
**melanophthalma_720**	*R. melanophthalma* C2	BRY C55056	USA, Wyoming, Johnson Co.: west of Buffalo	HM577277	44.33849	-106.7656	1581	SDL
melanophthalma_721	*R. melanophthalma* C2	BRY C55057	USA, Wyoming, Fremont Co.: Wind River Mountains	HM577278	42.73869	-108.8352	2122	SDL
haydenii_728	*R. haydenii*	BRY C55032	USA, WY, Sweetwater Co.; 16.1 E of Rason off Hwy 28 on Sqaw R	HM577301	-	-	-	SDL
haydenii_729	*R. haydenii*	BRY C55033	USA, WY, Sweetwater Co.; 16.1 E of Rason off Hwy 28 on Sqaw R	HM577302	-	-	-	SDL
novomexicana_730	*R. novomexicana*	BRY C55023	USA, Utah, Summit Co.: Ashley National Forest	KU934707	40.5976	-109.8406	2606	SDL, LLS, GS
novomexicana_731	*R. novomexicana*	BRY C55024	USA, Utah, Summit Co.: Ashley National Forest	HM577255	40.5976	-109.8406	2606	SDL, LLS, GS
**novomexicana_733**	*R. novomexicana*	BRY C55025	USA, Utah, Summit Co.: Ashley National Forest	HM577256	40.5976	-109.8406	2606	SDL, LLS, GS
melanophthalma_5165	*R. melanophthalma* C2	F Tshernyshev H2	Kazakhstan, Bektan-Ata	JX948269	47.4172	74.7933	700	S.E. Tshernyshev
melanophthalma_5166	*R. melanophthalma* C2	MS014620	Iran, East Azarbaijan, Jolfa, Daran Village	JX948276	38.807389	45.83151	1850	M. Sohrabi
**melanophthalma_5169**	*R. melanophthalma* C2	MS014623	Iran, East Azarbaijan, Jolfa, Daran Village	JX948273	38.807389	45.83151	1850	M. Sohrabi
parilis_6027	*R. melanophthalma* C4b	H9203310	Kyrgystan, Chatkal District - Chatkal Range	JX948192	41.53598	70.81087	2900	Lommi, S
parilis_6028	*R. melanophthalma* C4b	H9203313	Kyrgystan, Chatkal District - Chatkal Range	JX948193	41.53598	70.81087	2900	Lommi, S
melanophthalma_6724	*R. melanophthalma* C2	F Vondrak 9590	Czech Republic, Lovosice, Ceske Mountains	JX948285	50.848	14.45	-	J. Vondrak
**melanophthalma_6725**	*R. melanophthalma* C2	F Vondrak 9590	Czech Republic, Lovosice, Ceske Mountains	JX948286	50.848	14.45	-	J. Vondrak
melanophthalma_6742	melanophthalma	Fernandez-Mendoza s.n.	Chile, Region de Atacama, Paso de San Francisco	JX948289	-26.87	-68.31	4864	Fernandez-Mendoza s.n.
parilis_6743	parilis	Fernandez-Mendoza s.n.	Chile, Region de Atacama, Paso de San Francisco	JX948191	-26.87	-68.31	4864	Fernandez-Mendoza s.n.
porteri_6758	porterii (aff.) 'nevadensis'	Leavitt 12-611 (F)	USA, NV, Grant Range Wilderness Area-road to Timber Mountain	KU934702	-	-	-	-
polymorpha_6769	polymorpha	Leavitt 11-s.n. (F)	USA, UT, vicinity of Vernon Reservoir	KU934752	-	-	-	-
melanophthalma_6829	melanophthalma	Vondrak 9740	Turkey, Giresun, Giresun	KU934658	-	-	-	-
melanophthalma_6837	melanophthalma	Vondrak 9985 (PRA)	Russia, Altay	KU934659	-	-	-	-
**melanophthalma_6864**	melanophthalma	Vondrak 10035 (PRA)	Russia, Altay	KU934661	-	-	-	-
**melanophthalma_6891**	melanophthalma	KuvKz 1334	Russia, Orenburg	KU934662	-	-	-	-
melanophthalma_6892	melanophthalma	KuvKz 1335	Russia, Orenburg	KU934663	-	-	-	-
melanophthalma_6904	melanophthalma	Kaz 12940	Kazakhstan	KU934669	-	-	-	-
**melanophthalma_6905**	melanophthalma	Kaz 12922	Kazakhstan	KU934670	-	-	-	-
haydenii_8680	haydenii	Leavitt 8680 (F)	USA, Idaho, Lemhi Valley	KU934631	-	-	-	-
**haydenii_8681**	haydenii	Leavitt 8681 (F)	USA, Idaho, Lemhi Valley	KU934632	-	-	-	-
haydenii_8682	haydenii	Leavitt 8682 (F)	USA, Idaho, Lemhi Valley	KU934633	-	-	-	-
polymorpha_8663o	polymorpha	Leavitt-8663o (F)	USA, Utah, Duchesne Co., Nutter's Ridge	KU934754	-	-	-	-
porteri_8663r	porterii	Leavitt-8663r (F)	USA, Utah, Duchesne Co., Nutter's Ridge	KU934795	-	-	-	-
polymorpha_8663t	polymorpha	Leavitt-8663t (F)	USA, Utah, Duchesne Co., Nutter's Ridge	KU934756	-	-	-	-
shushanii_8664t	shushanii	Leavitt 13-TLM-001 (BRY-	USA, Utah, Wayne Co., Thousand Lakes Mountain	KU934860	-	-	-	-
**shushanii_8664u**	shushanii	Leavitt 13-TLM-001 (BRY-	USA, Utah, Wayne Co., Thousand Lakes Mountain	KU934861	-	-	-	-
polymorpha_8665l	polymorpha	Leavitt-8665l (F)	USA, Nevada, Nye Co., Barely Creek	KU934757	-	-	-	-
polymorpha_8665o	polymorpha	Leavitt-8665o (F)	USA, Nevada, Nye Co., Barely Creek	KU934758	-	-	-	-
porteri_8668c	porterii	Leavitt-8668c (F)	USA, Nevada, Nye Co., Mosquito Creek	KU934812	-	-	-	-
polymorpha_8935g	polymorpha	Leavitt 8935g (F)	USA, Idaho, Lemhi Valley	KU934773	-	-	-	-

Supplementary Figure 1

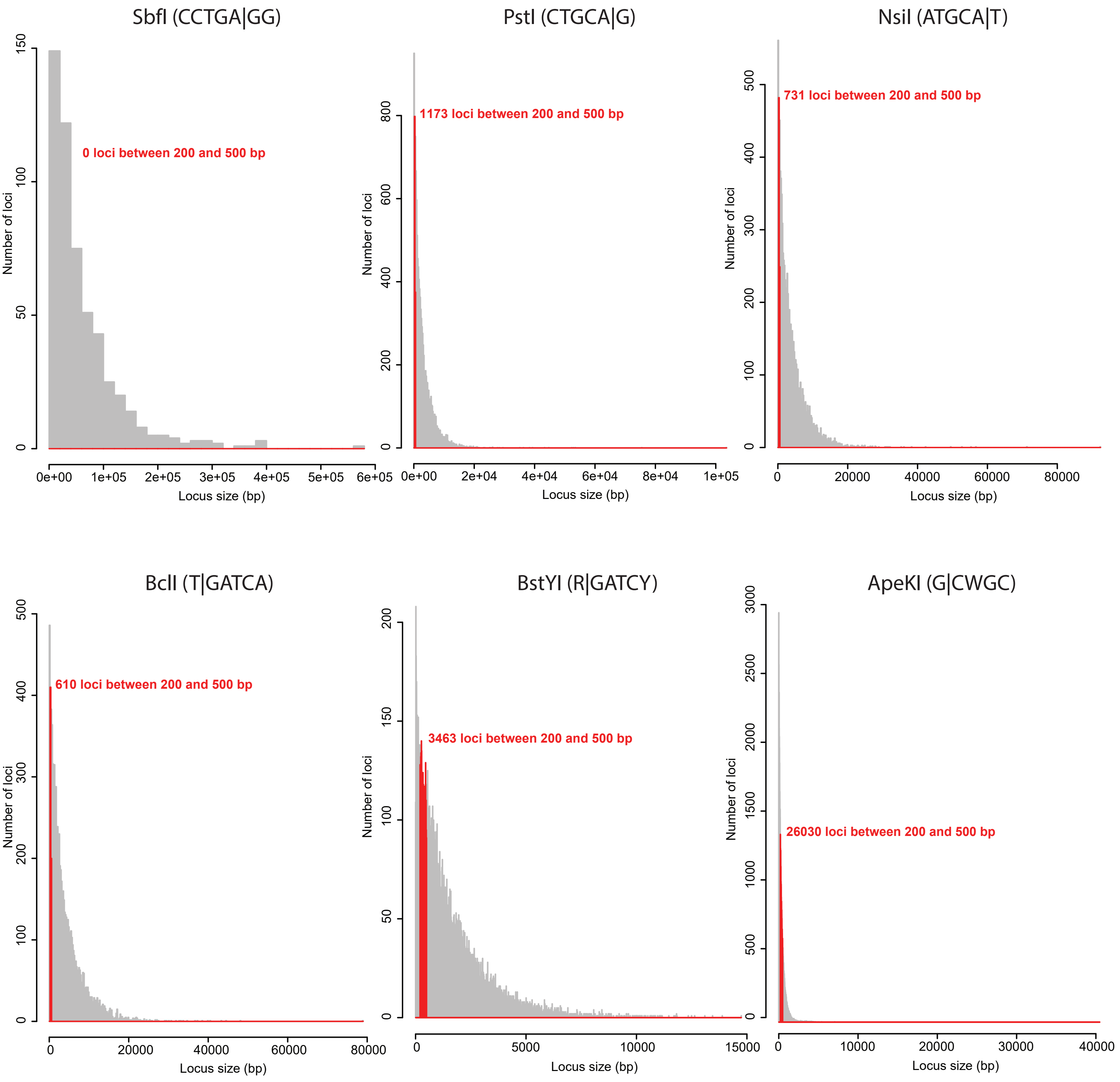

Supplementary Figure 2

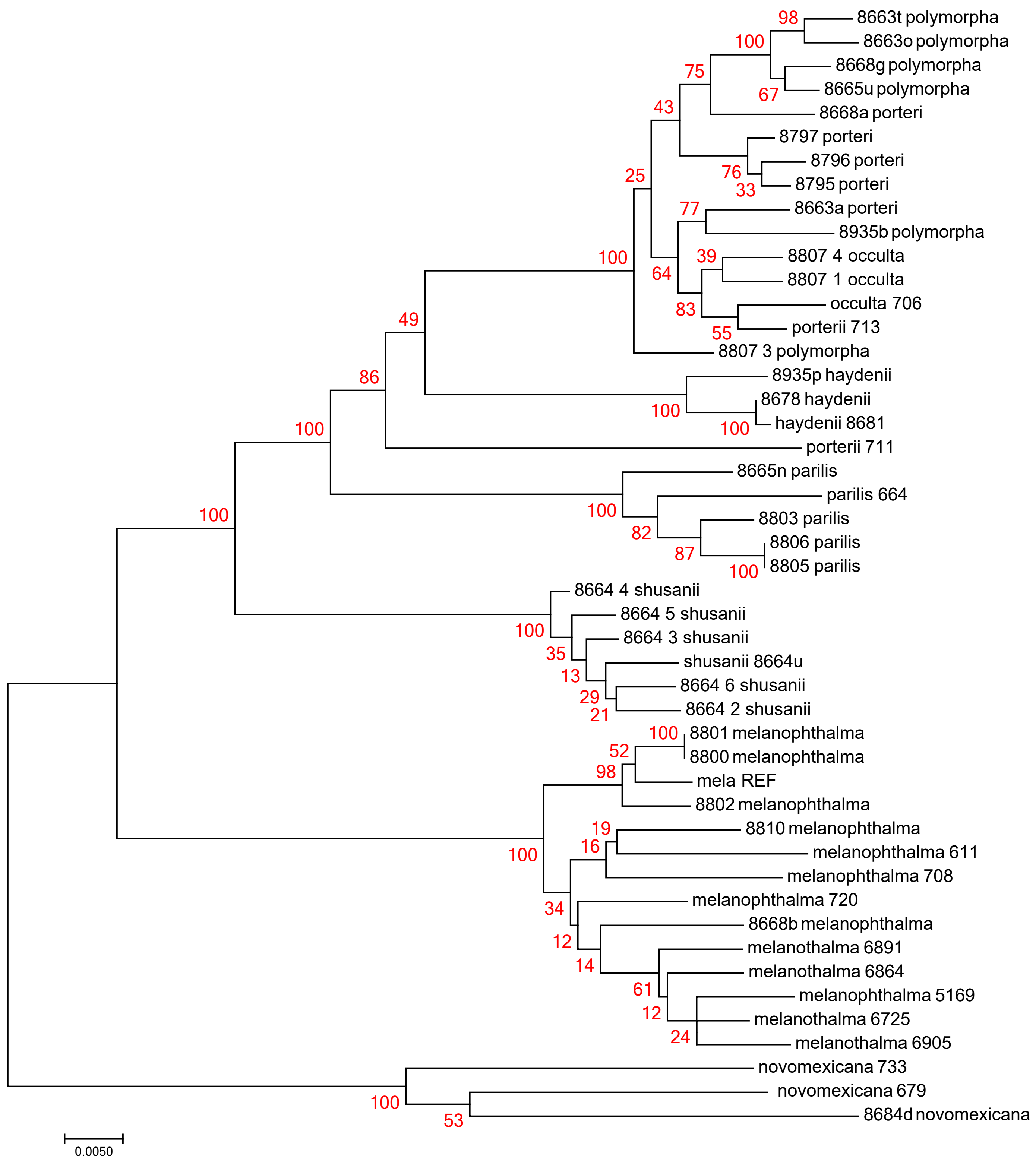

Supplement: Supplementary file 1 — Supplementary Material Online [file 41598_2017_9906_MOESM1_ESM.pdf]
